# A Cold Black Humour

**Published:** 2006-03-15

**Authors:** Lynne S. Wilcox

Robert Burton, an English Anglican priest, was one of the most educated and widely read men of his day. He lived from 1577 to 1640 and is remembered for his influential treatise, *The Anatomy of Melancholy* (1). Lacking knowledge of biophysical processes, early healers sought to develop models for the treatment of mental illness. In *Anatomy*, Burton reviews this history as well as the most modern theories of the 17th century. At that time, *melancholy* referred to all mental diseases and conditions and was considered one of four *humours* that controlled body systems: *blood* was a hot, sweet, red humour; *phlegm* was cold and moist; and *choler* was hot and dry. But *melancholy* was "cold and dry, thick, black, and sour, begotten of the more feculent part of nourishment, and purged from the spleen."

In the 17th century, treatment for an imbalance in melancholy might have included teas, infusions, or broths of laurel, white hellebore, bugloss, marigold, or pennyroyal — or even bloodletting with horse leeches. Burton discusses the debate on whether the brain, the heart, or some other body organ is the source of melancholy, then observes, "Our body is like a clock, if one wheel be amiss, all the rest are disordered." This metaphor remains apt for describing the role of mental health in overall wellness.

Modern integrative neuroscience demonstrates that the commonly accepted divide between mental and physical illnesses is a false dichotomy: almost all diseases are influenced by a combination of genomics, environment, and social conditions. Chapman et al have reviewed the epidemiologic association of depressive disorders, the most common forms of mental illness, with asthma, arthritis, cardiovascular disease, cancer, diabetes, and obesity (2). Kramer, in his comprehensive text *Against Depression*, discusses current supporting research in neurobiology (3). Chronic stress leads to the release of stress hormones that damage brain cells, especially in the hippocampus. The hippocampus regulates this hormone release, but as it becomes more damaged, regulation decreases, hormone release increases, and more brain cells are destroyed. This cycle affects organs throughout the body so that, for example, people with a history of depression have decreased bone density, increased risk of clotting, greater brain damage from stressors such as infections, higher rates of stroke, and higher rates of heart attack. Thus, there is both biologic and epidemiologic evidence to link environmental stress and depression to neurologic, hematologic, and cardiovascular disorders.

Even in modern times, the challenges of mental health have often been associated with silence and stigma. Families do not admit that one of their own is receiving treatment; few people reveal their diagnoses even to close friends. The Surgeon General's report on mental health (4) describes a comparison of population surveys conducted in the United States in the 1950s and the 1990s. Between those two periods of time, the public gained a better understanding of the causes of mental illness but continued to see people with such diseases as "socially undesirable" and "prone to violence." The discrimination was strongest against people with disorders that had not been explained by science. Even today, insurance coverage for mental health services is weaker than coverage for other health conditions, and people with mental illness may be barred from housing or employment opportunities.

Unfortunately, these disorders will not be healed by teas and infusions. Early screening, diagnosis, and treatment are critical tools for reducing severe morbidity and improving mental health. Encouraging mental health through primary prevention, however, remains a challenge. A few preventive measures are well recognized, such as the early treatment of syphilis or the removal of lead from paint, and more prevention options may be discovered as our understanding of the role of environment in mental illness expands.

The theme of this issue is mental health. We thank Dr James Lando, deputy associate director for science, National Center for Chronic Disease Prevention and Health Promotion, Centers for Disease Control and Prevention, for serving as guest editor for this issue. Public health professionals who emphasize primary prevention are better positioned to address the stigma of mental illness and the importance of mental health evaluation. Health education programs frequently use posters to teach the warning signs of cancer, diabetes, or heart attack. When posters displaying the warning signs for depression, addiction, or major mental illness appear with equal frequency, we will be on our way to conquering another group of ancient diseases.
